# Plasma Concentrations of Efavirenz and Nevirapine among HIV-Infected Patients with Immunological Failure Attending a Tertiary Hospital in North-Western Tanzania

**DOI:** 10.1371/journal.pone.0075118

**Published:** 2013-09-10

**Authors:** Daniel W. Gunda, Christa Kasang, Benson R. Kidenya, Rodrick Kabangila, Stephen E. Mshana, Jeremiah Kidola, Samuel E. Kalluvya, Gilbert W. Kongola, Hartwig Klinker

**Affiliations:** 1 Department of Internal Medicine, School of Medicine, Catholic University of Health and Allied Sciences, Mwanza, Tanzania; 2 Institute of Virology and Immunobiology, University of Würzburg, Würzburg, Germany; 3 Medical Mission Institute, Würzburg, Germany; 4 Department of Biochemistry and Molecular Biology, School of Medicine, Catholic University of Health and Allied Sciences, Mwanza, Tanzania; 5 Department of Microbiology and Immunology, School of Medicine, Catholic University of Health and Allied Sciences, Mwanza, Tanzania; 6 Mwanza Research Centre, National Institute for Medical Research (NIMR), Mwanza, Tanzania; 7 Department of Clinical Pharmacology, School of Medicine, Catholic University of Health and Allied Sciences, Mwanza, Tanzania; 8 Division of Infectious diseases, Department of Internal Medicine, University of Würzburg, Würzburg, Germany; University of Pittsburgh, United States of America

## Abstract

**Background:**

Sub-therapeutic and supra-therapeutic plasma concentrations of antriretrovirals are the significant causes of treatment failure and toxicity respectively among HIV-infected patients. We conducted this study to determine the pattern of efavirenz and nevirapine plasma drug concentrations among adult HIV-infected patients with immunological failure attending at a tertiary hospital in North-western Tanzania.

**Materials and Methods:**

A cross-sectional study was conducted among adult HIV-infected patients with immunological failure who have been on either efavirenz or nevirapine based antiretroviral regimen for more than 6 months. Patients were serially enrolled through routine Care and Treatment Clinic (CTC) activities. Plasma drug concentrations for efavirenz and nevirapine were determined by high performance liquid chromatography (HPLC) and Gas Chromatography (GC) respectively. Demographic, clinical and laboratory data such as viral load and CD4 counts were collected. Data analysis was done using STATA 12.

**Results:**

Of the 152 patients with immunological failure enrolled, the sub-therapeutic, therapeutic and supra-therapeutic plasma antiretroviral drug concentrations were found in 43/152 (28.3%), 76/152 (50.0%) and 33/152 (21.7%) respectively. Half of the patients were outside therapeutic window with either sub-therapeutic or supra-therapeutic plasma ARV drug concentrations. There was a significant difference in distribution of ARV adherence (*p*-value<0.001), NRTI backbone (*p*-value = 0.039), HIV stage (*p*-value = 0.026) and viral load (*p*-value = 0.007) within sub-therapeutic, therapeutic and supra-therapeutic ARV plasma drug concentrations.

**Conclusion:**

There is a wide inter-individual variability of plasma ARV concentrations among HIV patients with immunological failure, with a large proportion of patients being outside therapeutic window. This variability is significant based on ARV adherence, NRTI backbone, viral load and HIV stage. Routine therapeutic drug monitoring (TDM) could assist identifying these patients early and making timely correction to avoid virological failure, poor immunological outcome and prevent associated drug toxicities. Nonetheless, ARV adherence should be strictly emphasized on HIV patients with immunological failure.

## Introduction

The primary aim of antiretroviral therapy (ART) is to durably suppress the viral replication to undetectable levels to allow a satisfactory immune recovery [1], [2]. This is achieved by a long term use of ART at therapeutic concentrations [3]. An exposure of the virus at sub-therapeutic concentrations is likely to cause insufficient suppression of the virus and a probable selection of the resistant strains which will ultimately reduce the efficacy and durability of the ART [4]. Despite the overall success of ART, still many areas are reporting an inadequate virological suppression and drug toxicity complications [5]. The sub-therapeutic ART concentrations stand to be the main cause of poor therapeutic outcome [6]–[12]. On the other hand supra-therapeutic concentrations of ART are frequently associated with toxicities. Previous studies have indicated that between 30–40% of the patients assumed to have drug-related toxicities have abnormally high plasma concentrations of ARTs [4], [6], [13]–[17].

Efavirenz and nevirapine are the core and first line non-nucleoside reverse transcriptase inhibitors (NNRTI) commonly used to treat HIV infection [17]–[19]. Therapeutic drug monitoring (TDM) in patients on ART including these agents has suggested being beneficial in terms of efficacy and toxicity [17], [18]. TDM is an approach by which the course of therapy for a patient is guided by measurements of plasma drug concentrations enabling physicians to optimize ART drug effectiveness and to avoid drug-related toxicity [4], [20], [21]. A number of studies have documented a better treatment outcome in patients whose treatment was TDM guided than those whose concentrations were not monitored [22], [23]. In this regard TDM is useful in assessing adherence, investigating drug-drug interactions between antiretroviral drugs or with co-medications, preventing some ART drug toxicities, adjusting the dosage in particular populations, and increasing ART efficacy of some drugs in naive patients [4], [9], [12], [20], [22], [24]. TDM of ART agents could also be useful in provision of timely dosage adjustments to avoid drug sub-therapeutic or toxic concentrations [12], [22], [25]. Despite this fact TDM is not done in our setting and therefore the proportions of adult HIV patients with sub-therapeutic, therapeutic and supra-therapeutic ARV plasma concentrations are not known. Therefore it was the aim of this study to determine the pattern of plasma concentrations of the commonly used ART regimens containing efavirenz and nevirapine, among adult HIV-infected patients with immunological failure.

## Materials and Methods

### Study design and setting

This was a hospital based cross-sectional study which was conducted between April 2011 and March 2012 at Bugando Medical Centre (BMC) at Care and Treatment Center (CTC) in Mwanza, Tanzania. BMC is a 1000-bed capacity, tertiary and teaching hospital for the North Western Tanzania. The hospital serves around 13 million people and CTC activities is one of the core part of outpatient activities, which started in 2004, and currently it serves more than 10,000 patients, of whom about 3,600 are active on ARTs. The permission to conduct this study was obtained from the Catholic University of Health and Allied Sciences (CUHAS)/BMC joint ethics review board. The written consent was obtained from all participants.

### Study population, patients' enrolment and data collection

The study population was adult HIV patients on either efavirenz or nevirapine based regimen for more than 6 months with immunological failure attending BMC CTC. All patients were treated with the standard dose of nevirapine 200 mg twicw daily and efavirenz 600 mg once daily. Inclusion criteria were adult HIV positive patients diagnosed as per WHO guidelines, age over 18 years with immunological failure as per WHO guideline and still on first line. Pregnant women and patients co-treated with anti-tuberculosis medications and other concomitant medications known to interact with NNRTI [26] were excluded.

Immunological failure was diagnosed if the patient met one of the following criteria: i) persistent CD4 below 100 cells/mm^3^, ii) a drop of CD4 cell count below baseline pre-treatment level, or iii) a drop of CD4 cell count of 50% from peak on treatment value all in the absence of an ongoing co-infection and after a minimum of 6 months of ART. For criteria ii and iii, the CD4 cell count must also fall below 200 cells/mm^3^ to qualify as immunologic failure [27]. After giving consent a structured questionnaire was used to collect information regarding demographic data, body mass index (BMI), date of diagnosis of HIV, date of ART initiation, regime and adherence. ART adherence level in the last 30 days was assessed using pill counts [28]. The pill counts were performed by the study pharmacist, who counted the number of remaining pills at each drug refill visit. Pill count-based adherence was assessed using the formula [Adherence = (Number of pills dispensed - Number of pills returned) ×100)/(Number of pills prescribed daily×Number of days between pharmacy visits)]. Good adherence was defined as a value ≥95% pills whereas poor adherence was defined as a value <95%. The patients were instructed to have their medication at night and come the following morning for blood sample collection before taking their next ART dose. Two blood samples were drawn, one for viral load which was done at BMC main laboratory and the other sample was sent to Germany for TDM to determine the plasma concentrations of efavirenz and nevirapine.

### Sample collection, processing and analysis

Patients were instructed to have medication at night and come the following morning for blood sample collection before taking their next ART dose to determine their antiretroviral plasmatic trough concentrations for nevirapine and the mid concentrations for efavirenz. For each patient, 5 ml of whole blood was collected in plasma EDTA bottles for TDM, approximately 8 to 12 hours after the last dose of ART, just before the next dose was due. The samples were immediately centrifuged at 3000 rpm for 3 minutes to obtain plasma that was transferred into cryovials. The cryovials were stored at −20°C before shipment. The samples were packed and shipped to Germany in cold boxes with cooling packs maintaining a temperature of −30°C. The plasma concentrations of efavirenz (EFV) and nevirapine (NVP) were determined using High Performance Liquid Chromatography (HPLC) [HPLC Beckman Coulter System Gold] and Gas Chromatography (GC) [GC 6890; Agilent Technology] respectively as described previously [29], [30]. The well-established HPLC/GC method used in this study to determine plasma concentrations of non-nucleoside reverse transcriptase inhibitors (NNRTI) is highly specific and sensitive. The limit of detection (LOD) of nevirapine was determined at 2 ng/ml, the lower limit of quantification (LLQ) of nevirapine was reached at a concentration of 10 ng/ml. For efavirenz the LOD was 3 ng/ml, and the LLQ was 25 ng/ml. Additional 5 ml of whole blood was collected in a tube supplemented with EDTA (BD Biosciences) for plasma preparation and sent to BMC main laboratory for viral load analysis using COBAS AmpliPrep/COBAS TaqMan (Roche molecular systems, USA) according to manufacturer’s guidelines as described previously [31].

### Data management and analysis

Data were managed using Epi Data 3.1 (CDC Atlanta, US) and analysis was done using STATA version 12 (College Station, Texas, US). ARV drug concentrations were recorded as continuous variables. Based on the reference ARVs therapeutic ranges, defined as 1000–4000 ng/ml for EFV and 3400–8000 ng/ml for NVP [12], we defined 3 categories of ARV plasma drug concentrations: sub-therapeutic (below the lower therapeutic range limit), therapeutic (within the therapeutic range), and supra-therapeutic (above the higher therapeutic ranged limit) [12]. Categorical variables were summarized as proportion and their significance of the difference in distribution within the categories of ARV plasma drug concentrations was assessed using Pearson's Chi-square test or Fisher's exact test where appropriate. We used probability plots and Shapiro-Wilk normality test to assess the normality of continuous variables. Parametric continuous data were summarized as mean with standard deviation and the significance of difference in means within categories of ARV plasma drug concentrations was assessed using one way analysis of variance (ANOVA). Non-parametric continuous data were summarized as median with interquartile range and the difference in medians within the categories of ARV plasma drug concentrations was compared using Kruskal-Wallis equality-of-populations rank test. Inter-individual pharmacokinetic variability was evaluated through the coefficient of variation calculated as the quotient of the standard deviation divided by the mean plasma drug concentrations ×100. In determining the median and the inter-individual pharmacokinetic variability, patients with plasma drug concentrations below the lower limit of quantification of the assay (25 ng/ml and 10 ng/ml for efavirenz and nevirapine respectively) were arbitrarily considered as having a level of 24 ng/ml for efavirenz and 9 ng/ml for nevirapine. In all analyses the difference was considered significant if a p-value was less than 0.05.

## Results

A total of 152 HIV infected adult patients with immunological failure were enrolled in the study. Of these 79/152 (52.0%) were using nevirapine based regimen whereas 73/152 (48.0%) were on efavirenz based regimen. The ART regimens used were Zidovudine+Lamivudine+Nevirapine 46/152 (30.3%), Zidovudine+Lamivudine+Efavirenz 45/152 (29.6%), Stavudine+Lamivudine+Nevirapine 31/152 (20.4%), Tenofovir+Emtricitabine+Efavirenz 28/152 (18.4%) and Tenofovir+Emtricitabine+Nevirapine 2/152 (1.3%). The duration of use of these regimens ranged from 7 to 72 months. The mean age was 40.8±10.0 years with most patients 107/152 (70.4%) being females (Table 1). Of the 152 patients with immunological failure, 121/152 (79.6%) were in WHO clinical stage 2 or 3. Good adherence was observed in 84.2% (128/152) of patients while a viral load ≥400 copies/ µl was observed in 44.7% (68/152). There were 8/152 (5.3%) patients co-infected with either hepatitis B or C virus (HBV or HCV) infection, of these 7 had HBV and one had HBC. The median [interquartile range] plasma concentrations of efavirenz and nevirapine were 2112 [1349–3452] ng/ml and 4915 [2326–7044] ng/ml respectively (Table 1).

**Table 1:** Distribution of patients' characteristics among 152 participants.

Patient Characteristic	Number (%)/Mean±SD/Median [IQR]*
***Antiretroviral based regimen***	
Efavirenz	73 (48.0)
Nevirapine	79 (52.0)
*Mean age in years*	40±10.0
*Gender*	
Female	107 (70.4)
Males	45 (29.6)
***Median BMI in Kg/M^2^***	22.2 [20.5–24.8]
***Median antiretroviral concentrations (ng/ml)***	
Efavirenz	2112 [1349–3452]
Nevirapine	4915 [2326–7044]
***NRTI backbone***	
AZT+3TC	91 (59.9)
D4T+3TC	31 (20.4)
TDF+FTC	30 (19.7)
***Median Duration on ART in months***	40 [26–48]
***ARV Adherence level***	
Good	128 (84.2)
Poor	24 (15.8)
***Median Enrolment CD4 counts (cell/ µl)***	200 [133–288]
***Viral Load (copies/ µl)***	
≥400	68 (44.7)
<400	84 (55.3)
***WHO HIV stage***	
Stage 1	11 (7.2)
Stage 2	63 (41.4)
Stage 3	58 (38.2)
Stage 4	20 (13.2)
***Hepatitis B/C virus co-infection***	
No	8 (5.3)
Yes	144 (94.7)
***Plasma ARV Drug level***	
Sub-therapeutic	43 (28.3)
Therapeutic	76 (50.0)
Supra-therapeutic	33 (21.7)

* SD = Standard deviation; IQR = Interquartile range; CD4 = Cluster of differentiation; BMI = Body mass index; ARV = Antiretroviral; AZT = Azidothymidine (Zidovudine); 3TC = Lamivudine; TDF = Tenofovir; FTC = Emitricitabine; D4T = Stavudine.

Of the 152 patients enrolled, the sub-therapeutic, therapeutic and supra-therapeutic plasma antiretroviral drug concentrations were observed in 43/152 (28.3%), 76/152 (50.0%) and 33/152 (21.7%) respectively. Half of the patients were outside therapeutic window with either sub-therapeutic or supra-therapeutic plasma ARV drug concentrations. Based on the ARV regimens, sub-therapeutic plasma concentrations were more common among patients using nevirapine than those using efavirenz, 35.4% (28/79) versus 20.5% (15/73). Supra-therapeutic plasma antiretroviral drug concentrations were slightly lower among patients using nevirapine than those using efavirenz, 20.3% 16/79 versus 23.3% (17/73). These differences were not statistically significant (Table 2). Of the 43 patients with sub-therapeutic plasma antiretroviral drug concentrations, 17 (39.5%) had concentrations below the detection limit of the HPLC/GC. Of these, 12 were using efavirenz and 5 were using nevirapine.

**Table 2:** Comparison of distribution of patients' characteristics within plasma antiretroviral drug concentrations (sub-therapeutic, therapeutic and supra-therapeutic) among 152 participants.

PATIENT CHARACTERISTIC	PLASMA DRUG CONCENTRATIONS			*p*-value
	SUB-THERAPEUTIC	THERAPEUTIC	SUPRA-THERAPEUTIC	
	n = 43	n = 76	n = 33	
***Antiretroviral based regimen***				
Efavirenz	15 (20.5)	41 (56.2)	17 (23.3)	0.122
Nevirapine	28 (35.4)	35 (44.3)	16 (20.3)	
***Mean Age (years)***	38.3±10.4	40.8±9.7	44.0±9.8	0.053
***Gender***				
Female	34 (31.8)	50 (46.7)	23 (21.5)	0.311
Male	9 (20.0)	26 (57.8)	10 (22.2)	
***Median BMI (Kg/M^2^)***	22.9 [21.1–27.2]	22.1 [20.6–24.6]	21.6 [19.0–24.2]	0.299
***NRTI backbone***				
AZT+3TC	22 (24.2)	48 (52.7)	21 (23.1)	0.039
D4T+3TC	15 (48.4)	9 (29.0)	7 (22.6)	
TDF+FTC	6 (20.0)	19 (63.3)	5 (16.7)	
***Median ART duration (months)***	36 [24–48]	39 [27–48]	45 [33–48]	0.535
***ART Adherence***				
Good	26 (20.3)	70 (54.7)	32 (25.0)	<0.001
Poor	17 (70.8)	6 (25.0)	1 (4.2)	
***Viral Load*** (copies/ µl)				
<400	16 (19.0)	44 (52.4)	24 (28.6)	0.007
≥400	27 (39.7)	32 (47.1)	9 (13.2)	
***WHO HIV Stage***				
Stage 1 or 2	18 (24.3)	45 (60.8)	11 (14.9)	0.026
Stage 3 or 4	25 (32.1)	31 (39.7)	22 (28.2)	
***Hepatitis B/C virus co-infection***				
No	39 (27.1)	73 (50.7)	32 (22.2)	0.399
Yes	4 (50.0)	3 (37.5)	1 (12.5)	

* NRTI = Nucleoside reverse transcriptase inhibitor, ARV = Antiretroviral; BMI = Body Mass Index; AZT = Azidothymidine (Zidovudine); 3TC = Lamivudine; TDF = Tenofovir; FTC = Emitricitabine; D4T = Stavudine.

Sub-therapeutic drug concentrations were significantly more common (as supra-therapeutic was less common) among patients with poor ARV adherence, NRTI backbone comprising Stavudine+Lamivudine (d4T+3TC), advanced HIV stage and those with high viral loads than their counterparts. Generally, there was a significant difference in distribution of ARV adherence rate (*p*-value<0.001), type of NRTI backobone (*p*-value = 0.039), viral load (*p*-value = 0.007) and WHO HIV stage (*p*-value = 0.026) within the categories of ARV plasma drug concentrations (sub-therapeutic, therapeutic and supra-therapeutic). Table 2 summarizes the significance of the difference in distribution of various patients' characteristics within the categories of ARV plasma drug concentrations. The inter-individual variability was higher among patients using efavirenz based therapy than those using nevirapine based therapy (120.9% versus 88.7%). Generally, there was a wide inter-individual variability of plasma ARV concentrations among HIV patients with immunological failure using efavirenz and nevirapine in routine clinical practice as summarized in table 3.

**Table 3:** Inter-individual variability for Efavirenz and Nevirapine among 152 participants.

Antiretroviral drug	Number of patients	Mean plasmatic drug concentrations±SD in ng/ml	Inter-individual Coefficient of variation (%)
Efavirenz	73	3539.2±4831.5	120.9
Nevirapine	79	5448.7±4831.5	88.7

* SD = Standard Deviation

## Discussion

This study has demonstrated a presence of a wide inter-individual variability of plasma ARV concentrations among HIV patients with immunological failure in routine clinical practice, with a large proportion of patients being outside therapeutic window. This emphasizes that clinicians are often confronted with treatment failure or side-effects, and are in need of methods to evaluate drug exposure among these patients. The finding of higher inter-individual variability among patients using efavirenz based therapy than those using nevirapine based therapy was also observed in a study done in Italy [32]. However, our inter-individual variability was higher than that observed in Italy for both antiretroviral drugs (120.9% and 88.7% versus 85.1% and 50.1% respectively) [32].

In this study sub-therapeutic ARV plasma concentrations were detected in 28.3% of patients. Our findings are similar to that obtained in the study done in Netherlands among patients at a risk of treatment failure in a routine clinical care, in which 27.4% of the plasma concentrations were classified as having sub-therapeutic ARV plasma concentrations [21]. However, our findings are slightly higher than that from previous studies done in Uganda and Italy, in which the overall sub-therapeutic ARV concentrations were found in 14.3% and 16.9% respectively [33], [34]. This difference in prevalence might be due to the fact that in our study all participants had immunological failure, which was not the case in the Ugandan and Italian studies. Furthermore, our prevalence of sub-therapeutic ARV are lower than that from a study done British Columbia, in which the overall sub-therapeutic ARV concentrations were reported in 41.8% of patients with immunological failure [35]. This high prevalence could be attributed by the fact that all participants in the British Columbian study had a CD4 cell count less than 50 cells/ µl. On the other hand, supra-therapeutic ARV plasma concentrations were detected in 21.7% of patients. This prevalence is comparable to that reported from Uganda where 23.9% of the patients had supra-therapeutic ARV plasma concentrations [33], nevertheless all these observations embrace comparable consequence [17], [20] in clinical practice of HIV medicine.

The observations from this study are of paramount clinical relevance especially in resource-limited setting like ours. For the first time in Tanzania, we have demonstrated a presence of a wide inter-individual variability of plasma ARV concentrations and a significant association between good adherence and therapeutic ARV plasma concentrations among HIV-infected patients with immunological failure. We found that the proportion of patients with sub-therapeutic ARV plasma concentrations significantly increased with poor ART adherence, NRTI backbone comprising Stavudine+Lamivudine (d4T+3TC), increasing viral loads and advancing HIV stage. This finding is similar to that from previous studies done in Uganda and Italy [33], [34].

Therapeutic drug concentrations are a key to successful ART [7], [36], as any low drug concentrations observed in patients on ART has been extrapolative of a failure to achieve an immediate virological success and a longer term immunological failure [31], [37]. We found that the proportion of patients with sub-therapeutic ARV plasma concentrations was significantly high in patients with high viral loads (≥400copies/ µl) than those with low viral loads (<400copies/ µl) [39.7% versus 19.0%]. The presence of high rates of sub-therapeutic ARV concentrations among adult patients implies that these patients are standing a high risk of inadequate viral suppression and a subsequent potential of developing and accumulating resistant viral strains [4], [38], if these drug concentrations are not corrected timely [39]. On the other hand the patients with supra-therapeutic plasma NNRTI, are at a high risk of developing drug toxicity [16]-[18] which has also been reported as a common cause of non-compliance and discontinuation of their medications [6], [14]. Moreover it is well documented that drug toxicity happens commonly among patients with supra-therapeutic than among those with normal drug (therapeutic) concentrations [4], [6], [13], [14], [16], [17]. In this study 13.2% of patients with supra-therapeutic plasma drug concentrations also had high viral loads. This minor proportion of patients with supra-therapeutic and yet had high viral loads might be harboring HIV drug resistant strains. So both sub-therapeutic and supra-therapeutic ARV concentrations are clinically very important in the current era of HIV medicine. However this is a great challenge in Tanzania and other resource-limited settings where TDM is not done. Therefore, it is difficult to diagnose patients with sub-therapeutic and supra-therapeutic ARV status in order to make appropriate corrections to improve virological outcome of our patients. Since this study has demonstrated that a good adherence among patients with immunological failure is significantly associated with therapeutic ARV plasma level, strict emphasis on ARV adherence on this study population could be very helpful.

## Conclusion

There is a wide inter-individual variability of plasma ARV concentrations among HIV patients with immunological failure in routine clinical practice, with a large proportion of patients being outside therapeutic window. This variability is associated with ARV adherence, NRTI backbone, viral load and HIV stage. Routine therapeutic drug monitoring (TDM) could assist identifying these patients early and making timely correction to avoid immediate virological failure, long term poor immunological outcome and prevent associated drug toxicities. Good adherence is associated with therapeutic ARV plasma concentrations; therefore ARV adherence should be strictly emphasized on HIV patients with immunological failure.
